# A smart all-in-one device to measure vital signs in admitted patients

**DOI:** 10.1371/journal.pone.0190138

**Published:** 2018-02-12

**Authors:** Mariska Weenk, Harry van Goor, Maartje van Acht, Lucien JLPG Engelen, Tom H. van de Belt, Sebastian J. H. Bredie

**Affiliations:** 1 Department of Surgery, Radboud University Medical Center, Nijmegen, the Netherlands; 2 Radboud REshape Innovation Center, Radboud University Medical Center, Nijmegen, the Netherlands; 3 Department of Internal Medicine, Radboud University Medical Center, Nijmegen, the Netherlands; Osaka University Graduate School of Medicine, JAPAN

## Abstract

**Background:**

Vital sign measurements in hospitalized patients by nurses are time consuming and prone to operational errors. The Checkme, a smart all-in-one device capable of measuring vital signs, could improve daily patient monitoring by reducing measurement time, inter-observer variability, and incorrect inputs in the Electronic Health Record (EHR). We evaluated the accuracy of self measurements by patient using the Checkme in comparison with gold standard and nurse measurements.

**Methods and findings:**

This prospective comparative study was conducted at the Internal Medicine ward of an academic hospital in the Netherlands. Fifty non-critically ill patients were enrolled in the study. Time-related measurement sessions were conducted on consecutive patients in a randomized order: vital sign measurement in duplicate by a well-trained investigator (gold standard), a Checkme measurement by the patient, and a routine vital sign measurement by a nurse. In 41 patients (82%), initial calibration of the Checkme was successful and results were eligible for analysis. In total, 69 sessions were conducted for these 41 patients. The temperature results recorded by the patient with the Checkme differed significantly from the gold standard core temperature measurements (mean difference 0.1 ± 0.3). Obtained differences in vital signs and calculated Modified Early Warning Score (MEWS) were small and were in range with predefined accepted discrepancies.

**Conclusions:**

Patient-calculated MEWS using the Checkme, nurse measurements, and gold standard measurements all correlated well, and the small differences observed between modalities would not have affected clinical decision making. Using the Checkme, patients in a general medical ward setting are able to measure their own vital signs easily and accurately by themselves. This could be time saving for nurses and prevent errors due to manually entering data in the EHR.

## Introduction

The Early Warning Score (EWS) was developed in the United Kingdom in 1997 [1]. The EWS is a physiological scoring system that assists caregivers in detecting physiological changes and clinical deterioration in hospitalized non-critically ill patients [2, 3]. Since then, the EWS has been modified, which has resulted in the now commonly-used Modified Early Warning Score (MEWS). The MEWS includes systolic blood pressure (BP, mmHg), heart rate (HR, beats per minute), respiratory rate (RR, breaths per minute), temperature (Celsius), oxygen saturation (SpO_2_, %), amount of administrated oxygen (L/min), and the AVPU (Alert, responsive to Verbal stimulation, responsive to Painful stimulation and Unresponsive) [4–6]. A higher MEWS is associated with more ICU admissions and increased mortality [7–9]. Generally, the MEWS is determined for each patient at least three times per day to provide a general assessment of their clinical condition during hospitalization.

Although early warning scoring has been widely adopted and aims to create a safe, controlled situation, several issues have been raised about its practicability and efficacy. Measuring vital signs is time consuming, and frequently results in incomplete data input [4, 10]. A complete MEWS calculation takes approximately six minutes in total when accounting for measurements with several devices, data processing, and calculation of the MEWS. Inter-observer variation in measurements may exist, leading to a different MEWS in identical situations [11]. Further, results of the measurements are frequently entered in the Electronic Health Record (EHR) manually, and are therefore prone to mistakes [12, 13]. Finally, there is often no automatic alarm produced by the EHR to trigger a nurse to a higher level of surveillance or to call the Rapid Response Team (RRT). This makes MEWS monitoring rather subjective, and dependent on care professionals who may react differently to comparable situations.

**Fig 1:**
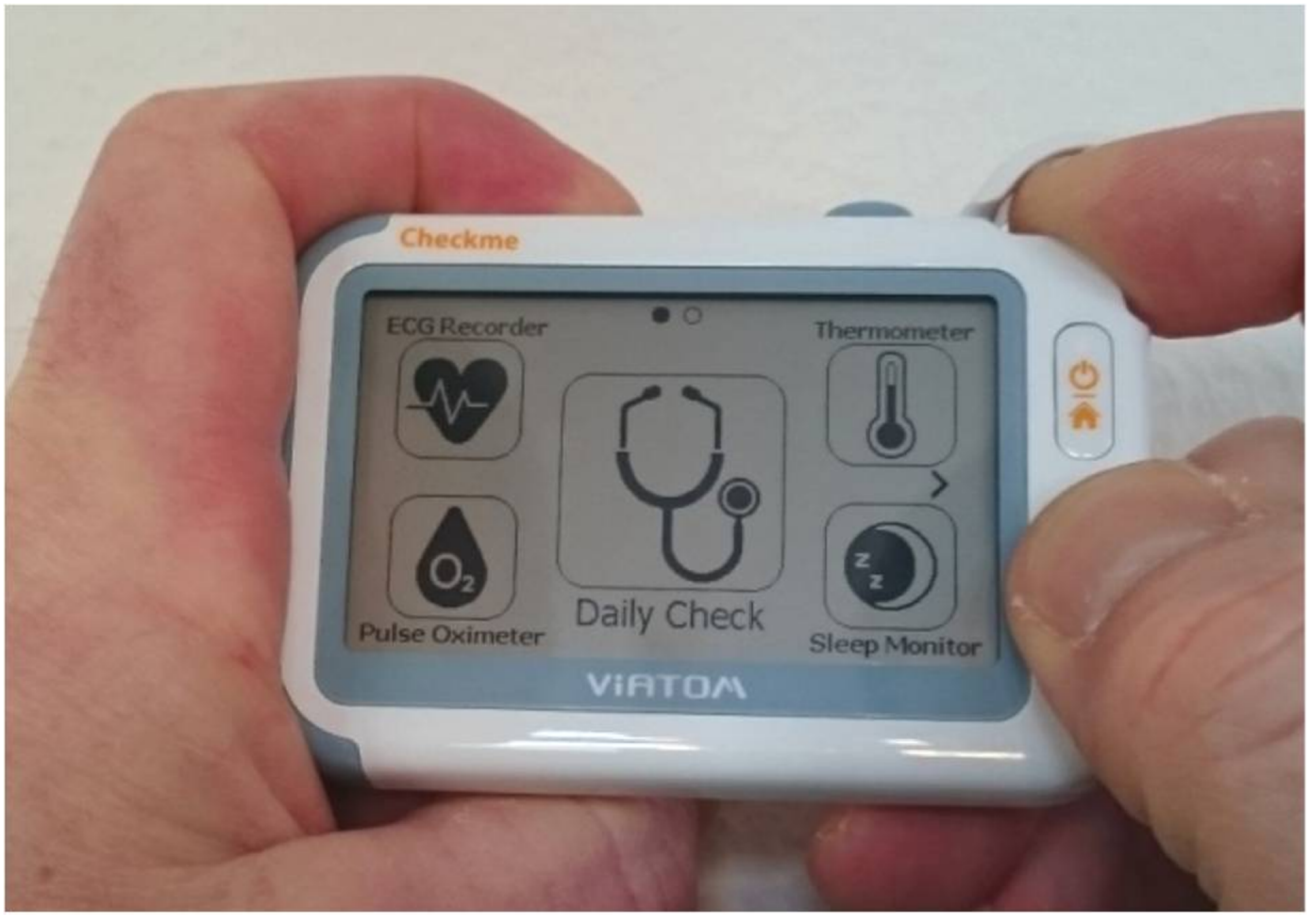
Viatom Checkme held in measuring position.

The Checkme Pro Health Monitor^™^ (Viatom Technology, Shenzhen, People’s Republic of China) is a newly released Conformité Européene (CE)-approved smart all-in-one device, which measures four of the five MEWS vital signs in less than 25 seconds (Fig 1) and can easily be handled by patients. Given its capabilities, the Checkme could represent a significant improvement in daily patient monitoring given its potential to reduce measurement time, inter-observer variability, and incorrect EHR inputs, without increasing costs. Moreover, the device enables patients to measure vital signs themselves, providing them greater insight into their health situation and increases patient empowerment in an in-hospital setting. Recent research showed promising results for BP measurements using the Checkme, however, evidence for its performance for other vital signs is limited [14].

In this prospective comparative study, we evaluated the Checkme for accuracy in assessing the individual vital signs used to calculate the MEWS in hospitalized non-critically ill patients on an Internal Medicine ward. Vital signs and calculated MEWS based on patient-operated Checkme measurements were compared with vital signs and calculated MEWS obtained by nurses and by a well-trained investigator (gold standard).

## Methods

### Setting and participants

Participants in this study were consecutive patients admitted to the General Internal Medicine ward of the Radboud University Medical Centre between March 2016 and May 2016. Patients were included if they were in a stable clinical condition, aged 18–75 years, mentally competent and able to understand instructions, and able to provide written informed consent. After reviewing the study protocol, the institutional review board waived the need for formal review and approval (local Ethical Committee Number 2016–2519).

### The Checkme

The study device, the Checkme, measures one or two lead ECG, body temperature, heart rate (HR), SpO_2_ and systolic blood pressure (BP) in a cuffless manner based on pulse transit time. The device also includes a pedometer and a sleep monitor. The Checkme has a “Daily Check” measuring mode, which measures all vital signs in less than 25 seconds. Before being able to conduct measurements with Checkme, a personal profile inclusive of gender, age, weight, and height is created on the device, and the systolic BP is calibrated once. This calibration is performed by measuring systolic BP simultaneously with a reference device, and by entering the reference systolic BP into Checkme. Systolic BP, HR, and SpO_2_ can then be measured by placing the right index finger beneath the lid on top of the device, the right thumb on the metal plate in the front, and the right middle finger on the metal plate on the back. Simultaneously, the metal plate on the left side of the device is then pressed against the palm of the left hand (Fig 1). Temperature can be measured separately via a sensor pressed against the forehead. The Checkme is not able to measure diastolic BP, RR or AVPU. To evaluate the results, data can be transferred via Bluetooth to a mobile phone or iOS/Android tablet with the Checkme app.

### Study procedures

After written informed consent was obtained, four measurement sessions were conducted in randomized order: a gold standard measurement in duplicate by a well-trained investigator, a measurement with the Checkme by the patient, and a regular vital sign measurement taken by a nurse. The gold standard measurements were performed to check for intra-observer variability. To obtain an accurate MEWS calculation from mixed data input, the investigator measurements were always carried out shortly before or after the Checkme measurement. The measurement sessions were conducted in the morning (6:30 AM), afternoon (2:00 PM) or evening (8:00 PM), always as close as possible to a regular nurse measurement, within a maximal time window of 30 minutes for all measurements. All measurements were done in the supine position in bed with patients in stable clinical condition. Patients were not allowed to leave their beds during the measurements. The investigator was blinded to the nurse’s measurement results to avoid confounding. Measured vital signs were HR, systolic BP, temperature, RR, SpO_2_, oxygen administration, and AVPU. A MEWS calculation was then performed according to established protocol. Gold standard and nurse vital signs were measured using an automated BP measuring device (Dinamap, GE Healthcare, Germany), a pulse oximeter (Dinamap, GE Healthcare, Germany) and a tympanic thermometer (Genius 2, Medtronic, USA). The BP calibration of the Checkme was conducted as a separate measurement in the morning, using the same Dinamap blood pressure measuring device. Following a calibration attempt, the device would display either “calibration succeed”, “calibration failed”, or “unstable measurement”. If the calibration failed or was unstable on three consecutive attempts, the patient was excluded from the study. Because the RR and AVPU cannot be measured with the Checkme, the values of the repeated investigator’s measurements were used for MEWS calculation in conjunction with Checkme vital signs.

### Methods of analysis

Statistical analysis was performed using Statistical Package for the Social Sciences (IBM SPSS Statistics version 20.0, SPSS inc., Chicago, Illinois, USA). The vital signs were described using mean with standard deviation (SD). Bland-Altman plots were created to assess intra-observer variability and differences in vital signs and calculated MEWS measured by the investigator, the nurse, and the patient (Checkme). In the plot, every data point represents the difference between two measurement methods. The solid line represents the mean difference, and the dotted lines represent the limits of agreement (1.96 SD). A one-sample t-test was performed on the difference between two measuring methods. A p-value < 0.05 was considered to be significant. For each vital sign, the clinically acceptable discrepancy between the three methods of measurements was predetermined. Clinically relevant differences were considered as follows: 5+ beats/min (HR); 5+ mm Hg (systolic BP); 0.5+°C (temperature); 2+ breaths/min (RR); 2+% SpO_2_. A difference in MEWS score of 1 point or more between different measurement sets was additionally considered to be clinically relevant.

## Results

### Study population

Fifty consecutive patients were included in the study for at least one set of vital sign measurements and MEWS calculations. Patients’ demographics and results of the Checkme calibrations are depicted in Table 1. In 41 of 50 patients (82%), initial calibration of the Checkme was successful and results were eligible for analysis. Two sets of measurements were performed in the morning (6:30 AM), 49 sets in the afternoon (2:00 PM) and 18 sets in the evening (8:00 PM). This resulted in a total of 69 measurement sets in 41 patients. Nine measurements performed by nurses (13.0%) were not complete (vital sign missing or not correctly entered in the EHR).

**Table 1:** Demographics of study population and results of calibration procedure.

		Total	Men	Women
**Gender (%)**		50 (100.0)	31 (62.0)	19 (38.0)
**Age (years)**		56.7 ± 15.8	58.7 ± 14.0	53.4 ± 17.8
**Weight (kg)**		79.5 ± 18.8	84.0 ± 16.5	72.2 ± 20.5
**Length (cm)**		171.9 ± 26.7	180.0 ± 7.1	158.7 ± 39.5
***Calibration of Checkme***				
**Successful calibration (%)**		41 (82.0)	25 (80.6)	16 (84.2)
**Number of successful attempts (%)**	1	30 (73.2)	18 (72.0)	12 (75.0)
	2	7 (17.1)	6 (24.0)	1 (6.2)
	3	4 (9.7)	1 (4.0)	3 (18.8)

### General results

Patient measurements using the Checkme took approximately 30 seconds per patient, and an additional 6–7 minutes were needed to calibrate the device. A successful first attempt BP calibration was obtained in 30 (73.2%) patients (Table 1). Repeated calibration attempts were needed in the other patients, and calibration eventually failed in 9 patients. Most failures were presumed to be due to shivering or cold hands. Calibration failure was not found to correlate with patient gender, age, or weight.

### Intra-observer variability

Table 2 depicts the vital signs and MEWS obtained via the well-trained investigator (gold standard) and the mean values of these measures in duplicate. Intra-observer variability was found to be significant for temperature measurements; measurements for other parameters were comparable for both attempts. Depending on the vital sign, 67.7 to 98.5% of the obtained results were less than the predefined clinically relevant differences. Sixty-two (91.1%) calculated MEWS measurements fell within the predefined limits of agreement.

**Table 2:** Mean values and differences for vital signs measured by gold standard, nurse and Checkme.

	Heart Rate (beats/min)	Systolic Blood pressure (mm Hg)	Temperature (°C)	Respiratory Rate (breaths/min)	Saturation (%)	MEWS
**Gold standard****:**						
**Mean of measure 1 and 2 ± SD**	74.1 ± 11.9	127.4 ± 17.4	36.9 ± 0.6	17.9 ± 4.2	95.9 ± 1.6	1.5 ± 1.4
**Measure 1 vs measure 2:**						
**Mean difference ± SD**^a^	-0.5 ± 3.8	0.4 ± 6.4	0.1 ± 0.3^b^	-0.2 ± 1.7	0.1 ± 0.9	0.1 ± 0.8
**Categories n (%)**	≤ 5: 59 (86.8)	≤ 5: 46 (67.7)	≤ 0.5: 65 (95.6)	≤ 2: 60 (88.2)	≤ 2: 67 (98.5)	≥+2: 5 (7.4)
	6–9: 8 (11.7)	6–14: 20 (29.4)	0.5–0.9: 1 (1.5)	3–4: 7 (10.3)	3–4: 1 (1.5)	0–1: 62 (91.1)
	≥ 10: 1 (1.5)	≥ 15: 2 (2.9)	≥ 1.0: 2 (2.9)	≥ 5: 1 (1.5)	≥ 5: 0 (0)	≥ -2: 1 (1.5)
**Nurse****:**						
**Mean ± SD**	73.5 ± 10.8	128.0 ± 18.3	37.1 ± 0.5	17.2 ± 2.2	96.3 ± 1.5	0.7 ± 1.1
**Nurse vs Gold Standard:**						
**Mean difference ± SD)**^a^	-0.8 ± 5.8	0.8 ± 9.7	0.2 ± 0.3^b^	-0.8 ± 4.3	0.4 ± 1.6	-0.8 ± 1.2^b^
**Categories n (%)**	≤ 5: 38 (65.5)	≤ 5: 27 (46.6)	≤ 0.5: 48 (82.8)	≤ 2: 27 (46.6)	≤ 2: 50 (86.2)	≥ +2: 1 (1.7)
	6–9: 16 (27.6)	6–14: 26 (44.8)	0.6–0.9: 9 (15.5)	3–4: 14 (24.1)	3–4: 7 (12.1)	0–1: 43 (74.1)
	≥ 10: 4 (6.9)	≥ 15: 5 (8.6)	≥ 1.0: 1 (1.7)	≥ 5: 17 (29.3)	≥ 5: 1 (1.7)	≥ -2: 14 (24.1)
**Checkme****:**						
**Mean ± SD**	74.6 ± 11.6	125.0 ± 19.2	35.7 ± 4.4	18.5 ± 4.5	96.8 ± 3.8	1.4 ± 1.6
**Checkme vs Gold Standard:**						
**Mean difference ± SD**^a^	0.7 ± 4.0	-2.7 ± 15.2	-0.7 ± 0.6^b^	n.a.	0.9 ± 4.2	0.1 ± 1.2
**Categories n (%)**	≤ 5: 57 (83.8)	≤ 5: 25 (36.8)	≤ 0.5: 25 (37.9)		≤ 2: 47 (69.1)	≥ +2: 8 (12.3)
	6–9: 10 (14.7)	6–14: 26 (38.2)	0.6–0.9: 24 (36.4)		3–4: 16 (23.5)	0–1: 55 (84.6)
	≥ 10: 1 (1.5)	≥ 15: 17 (25.0)	≥ 1.0: 17 (25.7)		≥ 5: 5 (7.4)	≥ -2: 2 (3.1)

^a^Positive (negative) value indicates higher (lower) mean value in measure 1 than in measure 2 of gold standard, and in nurse or Checkme measure than gold standard;

^b^p < 0.01 compared with gold standard

### Differences in vital signs

**Fig 2:**
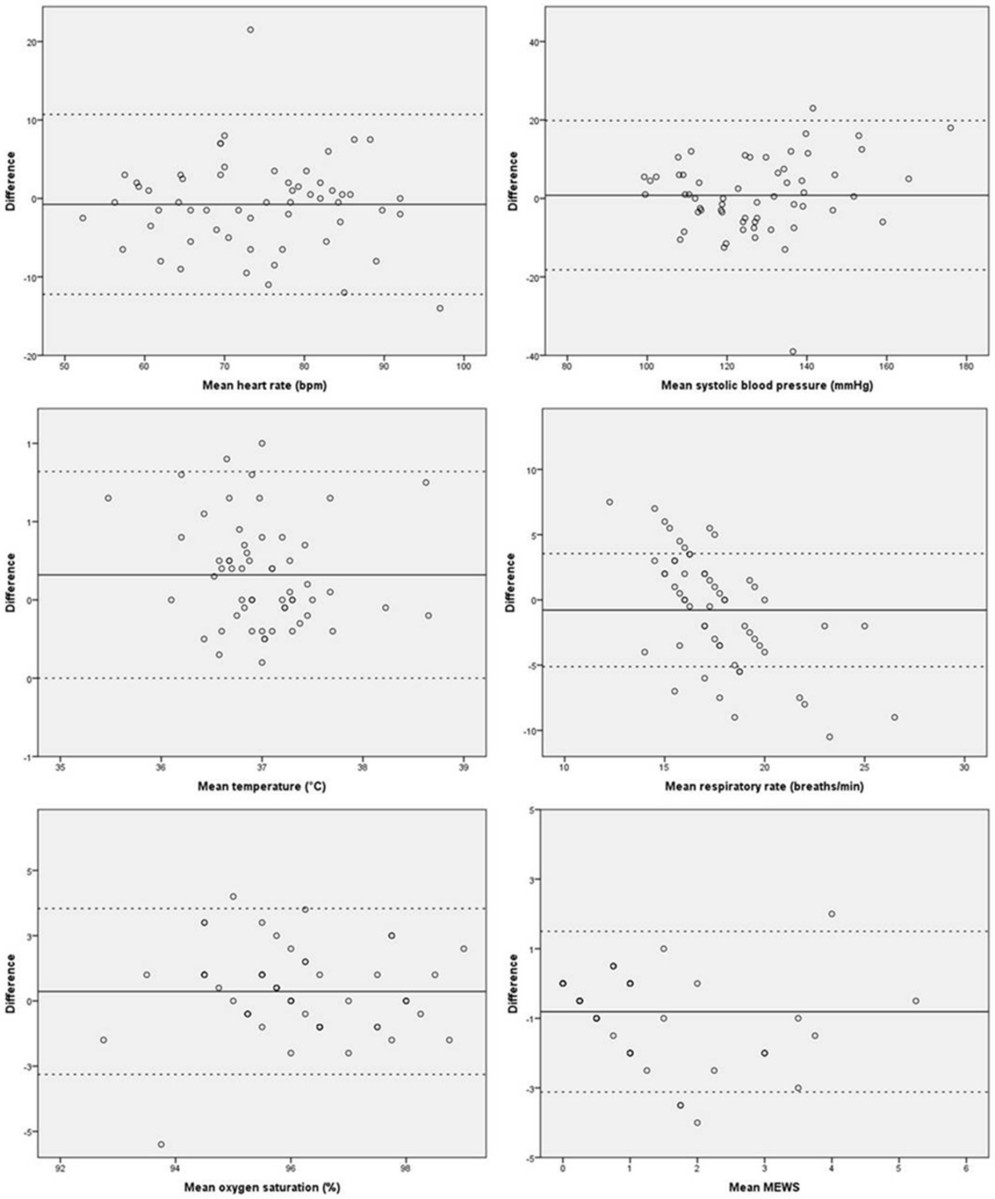
Bland-Altman plots of nurse and gold standard results.

Table 2 depicts the results of vital signs measured by nurses and patients (Checkme) in comparison with the gold standard. Data were equally distributed, and all mean differences were less the predefined clinically relevant limits for acceptable differences. Compared with the gold standard, the vital sign measurements recorded by the nurse showed a slightly but significantly higher temperature. Measurements of RR were additionally found to be somewhat discordant, with 14 (24.1%) of all measurements differing by 3–4 breaths/min and 17 (29.3%) of all measurements differing more than 5 breaths/min. Fig 2 shows the Bland-Altman plots of nurse and gold standard measurements.

**Fig 3:**
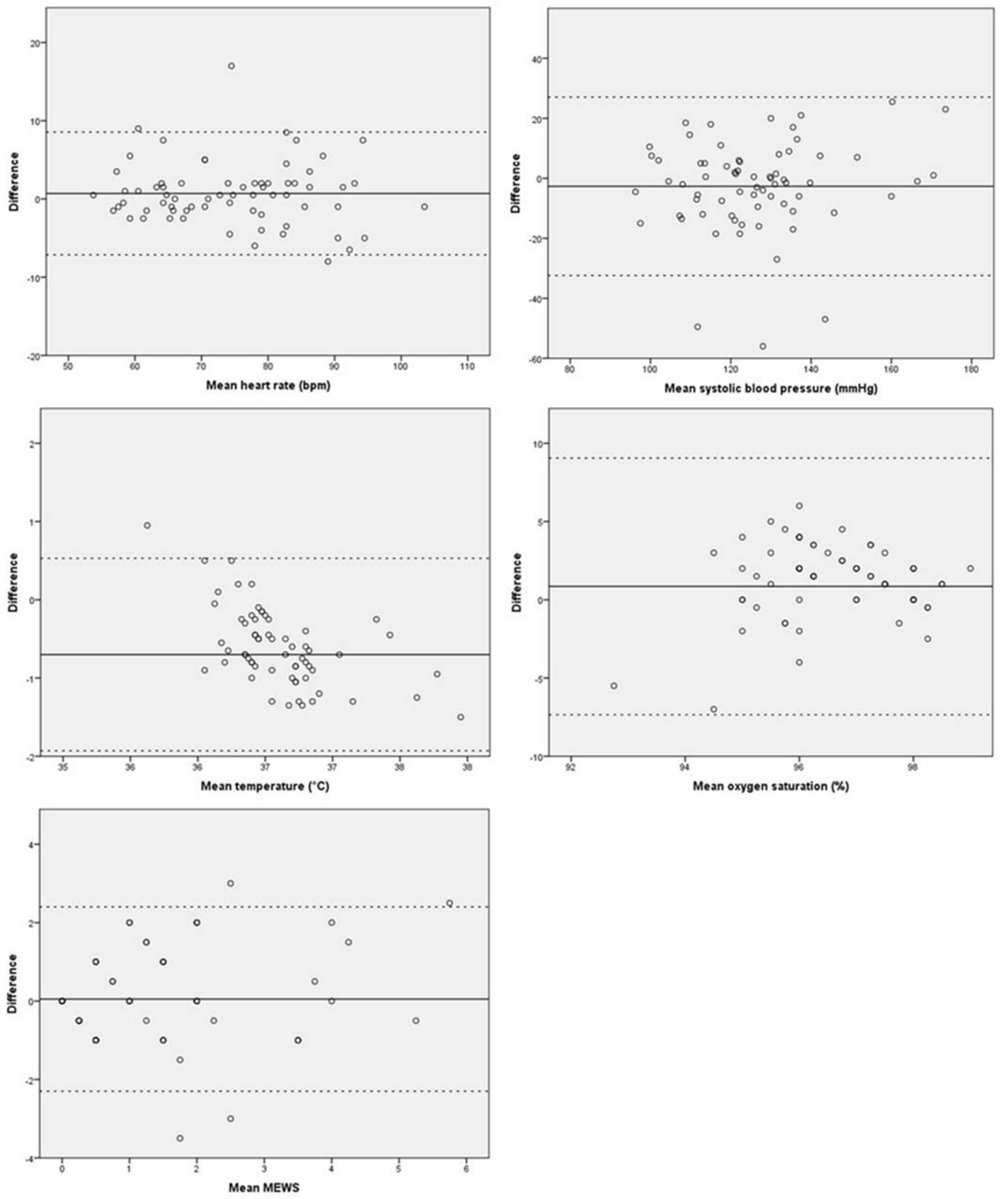
Bland-Altman plots of Checkme and gold standard results.

Fig 3 shows the Bland-Altman plots for Checkme in comparison with the gold standard. The results recorded by the Checkme for HR and SpO_2_ were largely in line with the gold standard measurements. Checkme temperature readings did differ significantly from the gold standard for temperature, with 17 (25.7%) of all measurements differing more than 1.0 °C from the gold standard. Further, for systolic BP, 17 (25.0%) of all measurements differed by more than 15 mmHg. Mean differences for all vital signs were, however, within the predefined limits of agreement.

### Differences in calculated MEWS

MEWS calculations on the basis of vital signs obtained by the gold standard measurements differed significantly from the MEWS based on nurse measurements, but were comparable to the MEWS derived from Checkme measurements. Compared with gold standard MEWS, the nurses’ MEWS differed by two points or more in 15 (25.8%) cases. By contrast, in 10 (15.4%) cases MEWS differed two points or more between gold standard MEWS and Checkme. Most MEWS calculations differed by 0–1 points between two methods. Mean differences of calculated MEWS were in range with the predefined accepted discrepancies. Three of 69 MEWS calculations by gold standard and nurses fell outside the limits of agreement (Fig 2). Bland-Altman plots are shown in Fig 3.

### Other

Patients reported their experiences about the use of the Checkme. In general, they found the device to be user-friendly, and described being able to measure their own vital signs with ease. Some elderly patients experienced difficulty holding the device firmly during measurement.

## Discussion

For the first time, the Checkme all-in-one device was tested in clinical practice in a significant number of hospitalized non-critically ill Internal Medicine patients to determine 4 of 5 vital signs necessary for early warning scoring. This study shows that after initial calibration of the Checkme to measure systolic BP, patients were able to easily and reliably measure their own vital signs. The results obtained by the Checkme were, to a large extent, comparable to the measurements obtained by nurses and by those of a gold-standard well-trained investigator. Measurement differences had a minimal effect on the aggregated MEWS.

Intra-observer variability between investigator measurements was low, supporting the use of this measurement as a “gold standard”. The differences in measured temperature between investigator measurements and between investigators’ and nurses’ measurements can be explained by the measuring error of 0.1°C of the tympanic thermometer used [15]. Significant differences for temperature were found between Checkme and the tympanic thermometer. Tympanic thermometers are often used in hospitalized patients, although the accuracy of tympanic temperature measurements for core body temperature measurement in the literature is mixed [16–18]. Checkme is able to measure infrared body temperature on the forehead and was recently validated [19]. A recent review by Geijer et al. showed that these methods of infrared body temperature measurement are not as accurate as invasive methods, but are comparable to tympanic thermometers [20]. Although absolute Checkme temperature measurements will be lower than core temperature measurements, the device is able to accurately monitor temperature changes in patients, which is often the primary finding of clinical interest [16].

Although more extensive differences were found for systolic BP measurements between gold standard and Checkme, these were not statistically significant. Checkme is able to measure BP without cuffs using pulse transit time, which is closely related to BP via cuff based methods and arterial compliance [21–23]. Although a validation study has yet to be published, BP measurement by the Checkme has been shown to be reliable and accurate in an earlier study [14]. Additionally, BP differences in our study had a minimal effect on differences in calculated MEWS, without important clinical consequences. Although the measurements were randomized, the gold standard and Checkme measurements were always undertaken directly after one another, whereas the nurse measurements sometimes had a time difference of 5 to 30 minutes before or after the other measurements. This could explain the differences between nurses’ and gold standard RR measurements. Inaccurate RR measurements by nurses and limited reproducibility as evidenced by significant inter-observer variability could further explain this discrepancy [24].

The calculated MEWS derived from Checkme values corresponded more closely with the gold standard MEWS than did the MEWS calculated by nurses’ measurements. The predominance of MEWS calculations by Checkme differed by one point or less from MEWS calculations obtained via the gold standard. Such differences had no important consequences for nurses’ actions, such as increased frequency of vital sign measurements, additional diagnostic procedures, or rapid response team calls.

An additional important underlying finding in this study is that conscious and non-critically ill patients were able to measure their own vital signs easily and in an accurate and reliable way when compared to nurses. Furthermore, the Checkme measurements by the patient took less time after BP was calibrated successfully, and patient comfort was enhanced by avoiding the need for cuff BP measurements. It is unknown whether the Checkme data would be more accurate if the nurse had performed the measurements using the device, as this was not the focus of our study.

There may be additional benefits to patient self-monitoring of vital signs. For example, in the home setting, patient self monitoring of BP has been shown to have a positive effect on BP regulation [25], and improves patients’ insight into their own health status and recovery [26]. Early experience with a device continuously monitoring vital signs resulted in increased interest in health data by patients on the internal and surgical ward (unpublished own data).

One drawback of the Checkme is the troublesome calibration of the BP measurement in approximately one-fifth of our patients. Our research group evaluated the performance of the BP monitor of the Checkme and also studied whether the position of the device influenced the outcome [14]. Twenty-five percent of the participants experienced difficulties during calibration in supine position. This percentage is higher, by contrast, than the 18 percent of patients in our study in whom most calibration difficulties were presumed to be due to shivering or cold hands. The troublesome calibration could limit home monitoring of vital signs by patients. It is expected that the next version of the Checkme will have a more simplified calibration procedure. Until then, the calibration procedure could be performed by trained physicians or nurses at the outpatient clinic, while the patient receives instructions about the use of the Checkme. Also, a trained physician or nurse will be availabe for patients in case of technical problems using the Checkme at home.

A strength of this study is the comparison of three measurement methods by an investigator, a nurse, and a patient, with blinding for the results of measurements. It is additionally important that we measured admitted patients in a clinical setting instead of healthy participants in a controlled setting. Time between gold standard and nurse measurements was mostly less than 10 minutes but could be 30 minutes. We cannot rule out slight changes in vital functions in a period of 30 minutes, however, due to the random order, the rigid protocol of nurse measurements in a patient group that is stable on the ward, comparison and interpretation of results seems justified. A limitation is that the Checkme is not able to measure diastolic BP and RR; RR is frequently used to inform clinical judgement in hospitalized patients [27]. Importantly, the next version of the Checkme will have the ability to measure RR. MEWS also includes oxygen administration and AVPU, which cannot be measured by the Checkme. Other EWS, such as the standardised early warning score, do not include oxygen admission but have still been shown to decrease inpatient mortality [28]. It could be possible that not all vital signs have a predictive value for clinical deterioration in different patient groups. Finally, critically ill or confused patients are not able to measure their own vital signs using the Checkme. Although the benefit of patients measuring their own vital signs is not attainable in this patient population, vital signs could still be collected reliably by nurses using the Checkme.

Future clinical research should focus on the use of Checkme and similar devices to predict clinical deterioration in various clinical settings, as well as patients’ and caregivers’ experiences using all-in-one devices. Furthermore, more frequent measurements and connections to hospital’s EHR, including automated alarming, may further increase patient safety during admission through earlier detection of clinical deterioration. The Checkme is suitable for home monitoring and is able to send all data to secured platforms via telemonitoring. Vital sign data could be used to optimize a patient’s home health or to identify underlying diseases such as atrial fibrillation prior to hospitalization and surgical procedures. It is expected that prehabilitated patients recover faster and with a lower complication rate postoperatively [29]. Cardiac patients could use the Checkme at home for 24-hour ECG registration and analysis, benefitting from a more comfortable method of monitoring than current holter monitors as well as from enhanced insight into their own health data.

In summary, our study demonstrates that patients in a general medical ward setting are able to measure their own vital signs easily and accurately by themselves, with comparable or even potentially superior accuracy to current nurse measurements. This could be time saving for nurses and prevent errors due to manually entering data in the EHR. While the rate of BP calibration failures limits Checkme applicability in certain patients at this time, it is anticipated that forthcoming versions of this device will address this shortcoming.
